# Telemedicine and Vascular Surgery: Expanding Access and Providing Care Through the COVID-19 Pandemic

**DOI:** 10.1177/00031348221109464

**Published:** 2022-10

**Authors:** Alina J. Chen, Savannah L. Yeh, Diana Delfin, Graciela Hoal, Natalie Barron, Toby Riedinger, Nika Kashanijou, Jessica Lieland, Katherine Bickel, Jessica B. O’Connell, Jesus G. Ulloa

**Affiliations:** 1 12222 David Geffen School of Medicine, University of California, Los Angeles, CA, USA; 2 Surgical & Perioperative Careline, Department of Surgery, Veterans Affairs Greater Los Angeles Healthcare System, Los Angeles, CA, USA; 3 Division of Vascular and Endovascular Surgery, David Geffen School of Medicine, University of California, Los Angeles, CA, USA

**Keywords:** vascular surgery, special topics, other

## Abstract

**Introduction:**

Access to surgical service is limited by provider availability and geographic barriers. Telemedicine ensures that patients can access medical care.

**Objective:**

The objective is to describe our use of telemedicine in delivering vascular surgery services to remote locations before and during the COVID-19 pandemic.

**Methods:**

We conducted a retrospective chart review analyzing care delivered at six vascular surgery telemedicine clinics over a 22-month period. We examined vascular diagnoses, recommended interventions, referrals placed, and emergency department visits within 30 days of evaluation. We calculated travel distance saved for patients between their local clinic and our main hospital.

**Results:**

We identified 94 patients and 144 telemedicine visits, with an average of 1.5 visits per patient (SD = 0.73). The most common referrals were for peripheral artery disease (20.2%) and abdominal aortic aneurysm (14.9%). Three patients were immediately referred to the emergency department due to concern for acute limb ischemia (2) or questionable symptomatic AAA (1). Telemedicine visit recommendations were distributed between no intervention (n = 30, 31.9%), medical management (n = 41, 43.6%), and surgical intervention (n = 23, 24.5%).

The surgical intervention cohort was most commonly referred to arterial revascularization (n = 4), venous ablation (n = 4), and arteriovenous fistula procedures (n = 4). Fourteen patients came to our main hospital for surgery and four to local providers. Average travel distance saved per telemedicine visit was 104 miles (SD = 43.7).

**Conclusions:**

Telemedicine provided safe, efficient care during the COVID-19 pandemic and saved patients an average of 104 travel miles per visit.

## Introduction

In 2019, the US Department of Veterans Affairs (VA) enacted the Maintaining Internal Systems and Strengthening Integrated Outside Networks (MISSION) Act to ensure Veterans have timely access to quality health care.^1^ The MISSION Act gives patients the option to receive care from providers in their local community if they meet certain accessibility criteria such as: the patient would have an extended drive time (30 minutes for primary care and 60 minutes for specialty care) or long travel distance to the nearest VA facility (40 miles).^1^

Patients living in remote or rural locations are known to experience disparities in care related to accessing specialty care.^2,3^ Moreover, Veterans using the VA health system have fewer financial resources, lower mean income, and if living in a rural area, have been demonstrated to use virtual care services with more frequency.^4^ In order to retain patients and increase access to care, the Veterans Affairs Greater Los Angeles Healthcare System (VAGLAHS) initiated a surgical telemedicine program focused on increasing access for Veterans living in remote locations.

As the telemedicine program was in its initial phases, the COVID-19 pandemic began and led to a drastic shift in medical care. Health care services needed to balance maintaining patient access to care while also minimizing patient and physician exposure to COVID-19. Veterans Affairs Greater Los Angeles Healthcare System adapted to employ our telemedicine program during the early phase of the pandemic to continue providing specialty care.

The objective of this study is to describe our use of telemedicine in delivering vascular surgery services to remote locations before and through the COVID-19 pandemic. We summarize referral questions, clinical care recommendations, surgical procedures performed within network or referred to community providers, and benefit to patients in terms of saved travel distance.

## Methods

The VAGLAHS offers services to 1.4 million Veterans residing in Southern California.^5^ Clinical sites include a tertiary care center located in West Los Angeles (WLA VA), 2 ambulatory care centers, and 8 outlying community-based outpatient clinics (CBOC).^4,6^ The vascular surgery department provides subspecialty care through telemedicine clinics between the tertiary care center (WLA VA) and 6 remote clinics in Bakersfield (107 miles from WLA VA), Lancaster (70 miles), Oxnard (55 miles), San Luis Obispo (181 miles), Santa Barbara (94 miles), and Santa Maria (151 miles). Telemedicine visits were conducted between an attending physician at the tertiary care center and an advanced practice provider present with the patient at the remote clinic site.

This study was a retrospective chart review of patient visit encounters completed through vascular surgery telemedicine clinics over a 22-month period. We examined presenting complaints, vascular diagnoses, imaging and tests ordered, recommended interventions, and referrals placed. In order to assess the safety of care delivered via telemedicine, we reviewed all emergency room (ER) visits including those outside the VA in the 30 days following each telemedicine appointment. Records of patient visits to ERs outside of the VA system are documented in the VA electronic medical record. We reviewed each ER visit to determine if the presenting condition was related to the care provided at the telemedicine visit.

We estimated the difference in travel distance for patients from their home zip-code to WLA VA compared to the distance to the nearest remote clinic using Google Maps (https://www.google.com/maps). We chose not to calculate travel time saved, as our tertiary care center is in a major metropolitan area with unpredictable traffic patterns. All analyses were conducted using Microsoft Excel (Microsoft, Redmond, Washington).

## Results

We identified 94 patients and 144 total vascular telemedicine visits over the 22-month study period. As summarized in Table 1, most patients were male (n = 91, 96.8%), with a mean age of 70 years. Patients attended an average of 1.5 telemedicine visits (SD = .73). The most common referral requests were for peripheral artery disease (20.2%), abdominal aortic aneurysm (14.9%), leg pain (13.8%), and post-operative follow-up (13.8%) (Table 1). Telemedicine providers treated patients with a wide range of vascular conditions summarized in Table 1.

**Table 1.** Study Cohort, Total N = 94.

Age, Avg (SD)	70 (9.9)
Gender, male, N (%)	91 (96.8)
Comorbidities, N (%)	81 (86.2)
Hypertension	67 (71.3)
Hyperlipidemia	64 (68.1)
Peripheral artery disease	32 (34)
Coronary artery disease	29 (30.9)
Diabetes mellitus	27 (28.7)
Chronic kidney disease	12 (12.8)
Deep vein thrombosis	5 (5.3)
Presenting conditions in telemedicine clinic	
Peripheral artery disease/claudication	19 (20.2)
Abdominal aortic aneurysm	14 (14.9)
Leg pain	13 (13.8)
Post-operative follow-up	13 (13.8)
Venous insufficiency	12 (12.8)
Lower extremity edema	11 (11.7)
Carotid stenosis	5 (5.3)
Wounds	5 (5.3)
Arteriovenous fistula	4 (4.3)
Lower extremity numbness	2 (2.1)
Subclavian stenosis	2 (2.1)
Anticoagulation management	1 (1.1)
Aortic ulcers	1 (1.1)
Deep vein thrombosis	1 (1.1)
Review abnormal studies	1 (1.1)
Splenic artery aneurysm	1 (1.1)
Transition of care	1 (1.1)

Avg = average.

SD = standard deviation.

Most patients (68%) presented with pertinent imaging completed prior to evaluation, 16% of patients had no vascular specific imaging completed prior to evaluation, and the remaining 16% did not require imaging prior to evaluation. Recommendations following telemedicine visit were distributed between no intervention (n = 30, 31.9%), medical management (n = 41, 43.6%), and surgical intervention (n = 23, 24.5%).

Among the 23 patients recommended surgical intervention, the most common were arterial revascularization (n = 4), venous ablation (n = 4), and arteriovenous fistula procedures (n = 4) including AVF construction and excision. Of note, 5 patients who were recommended surgical intervention did not undergo surgery within the study period. Two patients were lost to follow-up (one patient was recommended venous ablation, and one patient had superficial femoral artery in-stent stenosis), one patient was recommended aneurysm repair but elected to monitor with routine imaging, one patient was recommended venous ablation but elected for medical management, and the final patient had an incidental finding of renal cancer and was not medically optimized for surgery. (Table 2).

**Table 2.** Summary of Imaging Tests Ordered and Location Site Completed.

Imaging Type	WLA	CBOC	Total
ABI alone	2	4	6
Arterial ultrasound alone	0	1	1
ABI and arterial ultrasound	4	9	13
Ultrasound			
Arterial alone	0	1	1
Venous	17	3	20
Carotid	3	9	12
Aortic	3	2	5
Vein mapping	1	1	1
CT angiogram	15	6	21
CT abdomen/pelvis	4	2	6
Other^a^	0	2	2

^a^1 MRA, 1 MRI Spine.

Three patients were immediately referred from the telemedicine visit to the emergency department at our tertiary care center due to concern for acute limb ischemia (n = 2) and questionable symptomatic AAA (n = 1). One patient underwent superficial femoral artery recanalization with stent placement. One patient was admitted for lower extremity pain with ischemia but improved with systemic anticoagulation and revascularization was not pursued given his significant medical comorbidities. The final patient had concern for symptomatic abdominal aortic aneurysm but was found to be asymptomatic upon in-person presentation and subsequently discharged with plans for surveillance imaging. Of note, we did not identify any patients who had a vascular related emergency department visit within 30 days of the telemedicine appointment.

Eighty-nine imaging tests were ordered and completed as a result of their telemedicine visit; 49 were completed at the tertiary care center and 40 were referred to local imaging centers. The most common imaging tests were CT angiogram (22, 24.7%), venous ultrasound (20, 22.5%), and ABI and arterial ultrasound combined (20, 22.5%).

Patients saved an average of 104 miles (SD = 43.7) in travel distance per telemedicine visit.

Among patients who requested follow-up within their community, two patients already had established care with a local provider, and all others cited travel burden as the reason for not choosing to utilize our tertiary care center.

We anecdotally identified that having multiskilled teams were important during the early phase of the telemedicine program due to technical issues. Communication between the advanced practice provider and the vascular surgeon was crucial, as well as access to information technology services that could address and implement hardware and software issues as they arose. We observed that providers preferred complex patients with previous interventions be admitted to the main tertiary care center for expediting acquisition of vascular studies and providing care. Patients expressed being very grateful on many occasions for providers traveling to the local CBOCs and saving the patients a long drive.

## Discussion

Vascular surgery care, like many other specialties, is disproportionately concentrated in urban areas, making access for patients in rural communities difficult.^7^ Our experience using telemedicine for vascular surgery clinic has demonstrated that it is safe and results in significant travel distance savings. While our patients saved an average of 104 miles in travel distance, another VA health care system study showed savings of 145 miles.^8^ Not only does this save time, costs, and transportation wear and tear, but telemedicine provides a more patient-centered approach to vascular care and has the added benefit of decreasing the carbon footprint of the care we provide.

Literature on the use of telemedicine in vascular surgery care is limited. One study described the travel time, cost, and environmental emissions saved by vascular surgery patients using telemedicine services.^7^ While other surgical subspecialties have published literature on the use of telemedicine, our study presents data specific to vascular surgery and suggests that telemedicine can be successfully used to perform an initial evaluation, safely make assessments on the need for urgent or emergent care, and follow-up assessments of a wide range of vascular conditions.^8^

Our experience with telemedicine elucidated several interesting observations. First, having a provider physically present with the patient allowed for confirmation of the physical exam that was otherwise difficult to assess through a camera alone, such as pulse exam, Doppler signals, and skin findings of complex PAD patients. Communication between the advanced practice provider and the vascular surgeon was crucial, as well as having an efficient multiskilled team that was able to address and implement hardware and software issues as they arose. We also observed that providers preferred to have complex patients with previous interventions be admitted to the main tertiary care center for expediting acquisition of vascular studies. Interestingly, despite the distance to the tertiary care center, most patients still preferred to receive care within the VA system rather than being referred out to local community care.

Along with the benefits of telemedicine, there are still challenges that remain to be addressed. For example, despite providing patients closer clinic locations, we continued to experience a high no show rate of roughly 30-50%. One contributing factor to this high percentage might be that some patients reside in such remote locations that even a closer clinic was still a significant distance away. Additionally, patients had the option to participate in videos visits from their home, but the need for stable internet connection and a camera-enabled device remained a significant barrier to the provision of care over a virtual platform.

Our study has limitations that should be considered. We lack a matched control group to comparatively assess clinical outcomes, which we compensated for by using emergency department visits as a correlate of safety. Additionally, we did not formally document patient satisfaction using telemedicine, which could provide insight into how future appointments may be improved.

## Conclusions

Our experience using telemedicine for vascular surgery clinic has demonstrated that it is feasible, safe, and results in significant travel distance savings. We have received positive feedback from our Veterans regarding the improved access to subspecialty care through this program. We plan to continue with our telemedicine program and potentially expand to cover a broader geographic area and other modalities of telecare. Further investigation using standardized questionnaires to characterize patient satisfaction would allow us to analyze patient perspectives and preferences on the use of telemedicine.
